# Priority setting for mental health research in Chile

**DOI:** 10.1186/s13033-017-0168-9

**Published:** 2017-10-02

**Authors:** Pedro Zitko, Francesca Borghero, Cynthia Zavala, Niina Markkula, Emilio Santelices, Nicolás Libuy, Alfredo Pemjean

**Affiliations:** 1 Mental Health Department, Ministry of Health of Chile, Santiago, Chile; 2 Unidad de Estudios Asistenciales, Complejo Asistencial Barros Luco, Gran Avenida José Miguel Carrera 3204, San Miguel, 8900085 Santiago, Chile; 3 Faculty of Medicine, Universidad Diego Portales, Santiago, Chile; 4 Department of Public Health, Pontificia Catholic University of Chile, Santiago, Chile; 5 Facultad de Medicina, Clinica Alemana Universidad del Desarrollo, Santiago, Chile; 6 National Health Research Council, Ministry of Health of Chile’, Santiago, Chile; 7 Department of Psychiatry, Clinical Hospital of University of Chile, Santiago, Chile

**Keywords:** Mental health, Priority setting, Public health policies, Mental health research

## Abstract

**Background:**

Scientific knowledge is a fundamental tool for making informed health policy decisions, but the link between health research and public policy decision-making is often missing. This study aims to identify and prioritize a national set of research gaps in mental health.

**Methods:**

A multi-approach method to identify gaps in knowledge was developed, including (1) document analysis and identification of possible research questions, (2) interviews to Ministry of Health key informants, (3) focus groups with different stakeholders, and (4) a web consultation addressed to academics. The identified gaps were translated to a standardized format of research questions. Criteria for prioritization were extracted from interviews and focus groups. Then, a team of various professionals applied them for scoring each question research.

**Findings:**

Fifty-four people participated in the knowledge gaps identification process through an online consultation (n = 23) and focus groups (n = 18). Prioritization criteria identified were: *extent of the knowledge gap*, *size of the objective population*, *potential benefit*, *vulnerability*, *urgency* and *applicability*. 155 research questions were prioritized, of which 44% were related to evaluation of systems and/or health programs, and 26% to evaluation of interventions, including questions related to cost-effectiveness. 30% of the research questions came from the online consultation, and 36% from key informants. Users groups contributed with 10% of total research questions.

**Conclusion:**

A final priority setting for mental health research was reached, making available for authorities and research agencies a list of 155 research questions ordered by relevance. The experience documented here could serve to other countries interested in developing a similar process.

**Electronic supplementary material:**

The online version of this article (doi:10.1186/s13033-017-0168-9) contains supplementary material, which is available to authorized users.

## Background

Mental disorders are one of the principal causes of global disease burden. In 2010, they were responsible for 185 million disability-adjusted life years globally (7.6% of the total), the majority of them coming from developing countries [1]. The burden of disease due to mental disorders has increased by 38% in the last two decades, owing mainly to demographic changes [2]. In the case of Chile, mental disorders are particularly relevant, given that they cause approximately 18% of the national burden of disease, being the leading cause of all studied conditions [3].

Scientific knowledge is a fundamental tool for making informed health policy decisions, for decreasing burden of disease, and achieving universal coverage. This is especially important in a context of limited resources [4].

In 2009, the total investment in health research reached US$240 billion, 90% of which was carried out in high-income countries and, at least in the publication of clinical trials, focused on health problems relevant for those countries [5]. Furthermore, a report of the Commission on Health Research for Development from 1990 for the first time presented the concept of the 10/90 gap, a well-known indicator of disparity, which states that only a small proportion of investment in health research is spent on diseases affecting low- and middle-income countries [6].

Similarly in the mental health area, 95% of publications come from high-income countries, and less than 1% from low-income countries, a tendency that has remained stable in the last few decades [7].

In light of this scenario, influential international entities dedicated to public health are calling for more research, identifying it as a component of national health systems [8–10]. These groups emphasize the importance of aligning investigation with knowledge gaps in each country, in order to move research from the academic world toward public health programs close to the demand and provision of health services [4, 11]. This aligning process reaches special importance in the field of mental health, where the determinant factors are more context-dependent than in other health problems [11].

To achieve the aforementioned, procedures are needed to identify and prioritize knowledge gaps present in health decision-making [12]. Such exercises have been developed at global, regional, and country levels [13].

To date, few low- and middle-income countries have developed their own practices of priority setting for health research [14, 15]. In the countries that have done so, these processes often present methodological limitations, as well as scarce linkage with health public policy decision-making [14, 16]. An example of similar efforts from high-income countries, the European ROAMER program identified six mental health research priority areas for 27 European countries using a combination of systematic mapping of the literature, expert workshops and surveys with input from more than 1000 expert researchers and stakeholders [17].

Chile does not have an established periodical process to identify and prioritize health research needs. Consequently, a prioritization process specifically for mental health is also needed. The purpose of this study is to identify and prioritize a national set of research gaps in mental health in Chile, and to document the prioritization process.

## Methods

The selection of research priorities was performed in two stages. The first stage consisted of four strategies to identify knowledge gaps: document analysis, interviews, focus groups, and an online consultation of the scientific community.

**Fig. 1:**
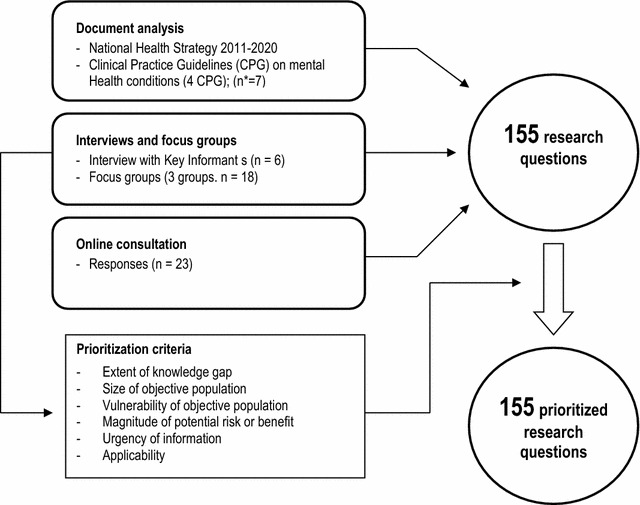
Identification and prioritization of knowledge gaps relevant to sectoral and intersectoral mental health policy decision-making

The second stage considered the elaboration and later application of prioritization criteria for ranking each knowledge gap (i.e. research question). Figure 1 shows a general outline of the study. Data collection was carried out between August 2010 and April 2011.

### Document analysis

The first analyzed document was a chapter from the National Health Strategy (NHS) to Meet Health Objectives for the Decade 2011–2020 [18], related to the reduction of disability associated to mental disorders. Knowledge gaps were identified by revising the justification of the underlying assumptions that supported the sections of diagnosis and strategies of the text. Two persons carried out the revision, and the results were presented and agreed upon by a team of professionals from the Department of Mental Health of the Ministry of Health (DMH, n = 7 people).

The second group of documents corresponded to Clinical Practice Guidelines, which are elaborated by the DMH and were updated during this process. Four out of five national mental health guidelines existing at the time were reviewed: Depression in Adults, Depression in Adolescents, Bipolar Disorder, and Harmful Alcohol and Drug Use in Adolescents. In addition, these documents are part of the Explicit Guarantees in Health (GES) program, and as such binding to clinicians attending patients in the program. In each guideline, knowledge gaps relevant for the formulation of recommendations were identified. The only guideline that was not reviewed as part of the process was the one on schizophrenia, because that guideline had recently been updated.

### Interviews and focus groups

Semi-structured interviews were carried out with six key informants from the Ministry of Health, selected by the hierarchy and participation level in mental health decision-making process: Heads of the Divisions of Health Planning; Disease Prevention and Control; Primary Care; and Mental Health, along with the Coordinator of the Unit of Mental Health Care Networks, and a consultant for the DMH.

To capture the knowledge gaps identified by groups outside the Ministry of Health, three focus groups were held: one with representatives from groups of mental health service users (n = 6), the second with members of the National Commission for the Protection of Persons with Mental Disorders (NCPPMD) (n = 5), and the third with clinical services staff members (n = 7). All the groups of mental health service users, with which the Ministry has formal contact, were invited, as well as all members of the NCPPMD. In the third group, selection of participants was by convenience. Everyone invited in the interviews and focus groups accepted to participate.

Two facilitators were present in the individual and the group interviews. One directly guided the discussion, while the other recorded the participants’ information and the main points addressed in the interviews. Each interview was recorded to back up the collected information. For both types of interviews, the interview guide considered three phases: 1. An awareness exercise, adapted to each individual or group; 2. The proposal of significant knowledge gaps, related to mental health decision-making; and 3. Identification of prioritization criteria. Each individual interview lasted between 20 and 60 min, approximately, and the focus groups, which were attended by 3 to 10 informants, between 2 and 3 h. The translated interview guide used for semi-structured interviews and focus groups. In each interview and focus group, small changes in the language and the awareness exercise were made depending on the group (patients, clinicians, health managers).

### Online consultation and categorization

A fourth strategy was specifically designed to capture the knowledge gaps identified by academics and researchers involved in mental health. An online platform was created and an invitation to participate was extended via email, explaining the purpose of the consultation and providing a link to the platform. The invitation was sent to a list of 63 academics, previously identified by the DMH as being active researchers in the field of mental health in Chile. Then they were asked to disseminate the invitation to their colleagues and other potentially interested individuals. In total, 23 academics answered the questionnaire.

Access to the platform required the participants’ identification and institutional affiliation, and then they were asked to formulate a research question, using a pre-established format, and categorize their question in one of the following areas: (1) Basic Sciences, (2) Natural History of Disease/Epidemiology/Risk Factors/Social Determinants, (3) Evaluation of Interventions (effectiveness, cost-effectiveness, etc.), (4) Evaluation of Health Systems and/or Programs, and (5) Evaluation of Intersectoral Public Policies.

These categories were adapted from similar prioritization processes, such as the Child and Nutrition Research Initiative [19], and have also been described elsewhere in scientific literature [12, 20]. The research questions included in the present study correspond to those received during the first 15 weeks the online platform was operational.

### Formulation of questions

All of the knowledge gaps and research questions were reviewed and adapted to a standard format, which included: exposed population, intervention or exposition, comparison, and result or outcome. The questions were categorized according to source and area, using the same categories as the online platform.

### Prioritization

The questions were ranked using the criteria identified during the interviews and focus groups, namely: (1) extent of the knowledge gap (2) size of the target population (3) magnitude of the potential benefit (4) vulnerability of the objective population (other than from the mental health condition) (5) urgency for policy making in reaching the gap, and (6) applicability (Additional file 1: Table S1). Each criterion was divided into a three-point scale. The questions were scored by a team made up of DMH professionals and two consultants—an epidemiologist with mental health experience and a psychiatrist with public policy training.

The final score of each research question was calculated by summing the scores of the criteria, which received equal weight. Since the nature of some of the research questions prevented the use of all criteria (e.g. questions of prevalence), the final scores were standardized from 0 to 100, where a higher value signifies greater priority.

Data analysis consisted of calculating the frequency of research questions stratified by source and area. Absolute numbers were preferred for simplicity. The knowledge gaps were ordered by priority score.

### Ethical aspects

This study did not involve human subject interventions. Informed consent was obtained before carrying out and recording the interviews and focus groups. Electronic records of the interviews and focus groups were eliminated once their content was extracted. An abbreviated report of the results was sent to each participant.

## Results

In total, 54 people (47% women) participated directly in the data collection process, and 155 knowledge gaps were identified (Additional file 1: Tables S1–S3).

Table 1 presents the distribution of questions according to the research area category and source. The majority of the questions (44%) were concentrated in the category Evaluation of Systems and/or Health Programs, and no questions were identified in the area of Basic Sciences. The online platform provided the greatest number of research questions (30%). The groups comprised of mental health service users and members of the NCPPMD provided 10% of the research questions.

**Table 1:** Distribution of identified research questions by area and source categories

Category/source	Document analysis		Interviews and focus groups				Online consultation	Total
	National Health Strategy	Clinical practice guidelines	Key informants	Users	NCPPMD^a^	Directors of Clinical Services		
Basic sciences	0	0	0	0	0	0	0	0
Natural history of disease/epidemiology/risk factors/social determinants	7	0	8	0	0	8	6	29
Evaluation of interventions (Effectiveness. Cost-effectiveness, etc.)	3	8	8	4	2	6	10	41
Evaluation of systems and/or health programs	8	0	20	3	4	8	25	68
Evaluation of intersectoral public policies	4	0	5	2	0	1	5	17
Total	22	8	41	9	6	23	46	155

^a^ *NCPPMD* National Commission for the Protection of Persons with a Mental Disorder

Content from the clinical practice guidelines was used to formulate questions largely related to the validation of screening and follow-up instruments for mental health conditions, categorized as Evaluation of Interventions.

The category Evaluation of Intersectoral Public Policies contained only 11% of the research questions, which came primarily from key informants of the Ministry of Health and the online consultation.

Table 2 shows the 10 questions with the highest scores, indicating the category and sources. However, because there were four questions sharing the same score, in fact 13 questions are presented. Most of the questions with the highest prioritization scores were in the category Evaluation of Systems and/or Health Programs, and they were principally identified within the National Health Strategy (NHS) content or by key Ministry of Health key informants.

**Table 2:** Prioritized research questions for decision-making on sectoral and intersectoral public policies related to mental health, with the highest scores

Question	Source	Category	Score
What are the determining factors of the variability of the performance of human resources in primary health care in the detection and management of people with mental disorders?	Key informant	Evaluation of System and/or Health Program	83.3
What are the determining factors of the variability of the comprehensive mental health care program, between primary care centers?	NHS	Evaluation of System and/or Health Program	83.3
What are the determining factors of the unchanging and unequal geographic distribution of specialized mental health human resources?	Users	Evaluation of System and/or Health Program	75.0
What is the effective coverage of interventions to manage mental disorders in affected individuals?	NHS	Evaluation of System and/or Health Program	70.0
What is the variability of the performance of the psychologists in the comprehensive mental health care program in primary care?	NHS	Evaluation of System and/or Health Program	70.0
What is the prevalence of mental disorders in minority populations (ethnic, immigrants, homeless)?	NHS	Natural History of Disease/Epidemiology/Risk Factors/Social Determinants	70.0
What are the determining factors of the performance of the mental health services of the different levels of care?	Key informant	Evaluation of System and/or Health Program	66.7
What is the effectiveness of the community model of mental health care, compared to the traditional care model in the general population?	NHS	Evaluation of System and/or Health Program	66.7
What are the determining factors of the performance of the community model in different mental health care facilities?	NHS	Evaluation of System and/or Health Program	66.7
What are the determining factors of compliance to the Clinical Practice Guidelines (CPG) of schizophrenia, depression, and harmful alcohol and drug use?	NHS	Evaluation of System and/or Health Program	66.7
What is the cost-effectiveness ratio of the psychosocial activities in municipal, educational, and work settings in the general population?	Key informant	Evaluation of Interventions	66.7
What is the incremental cost-effectiveness ratio of evidence-based interventions to prevent adolescent suicide?	Online consultation	Evaluation of Interventions	66.7
What are the transgenerational factors associated with intrafamilial child sexual abuse?	Directors of Clinical Services	Natural History of Disease/Epidemiology/Risk Factors/Social Determinants	66.7

The prioritization criteria and full list of 155 prioritized questions are provided as Additional file 1: Tables S1–S2.

## Discussion

Currently in Chile and other low- and middle-income countries, there is no clear and operative link between health research and public policy decision-making. In this context, the present study represents an important step forward. Through a systematic, transparent, and participatory process, we have identified and prioritized 155 knowledge gaps relevant to health policy decision-making, which, for the first time in Chile, focused specifically on mental health topics.

Other research groups around the world have carried out similar prioritization exercises in mental health. Tomlinson et al. applied the methodology of the Child and Nutrition Research Initiative (CHNRI) and identified 55 global mental health research questions. Unlike our study, their questions were proposed only by experts (n = 39, 74% psychiatrists), some of who also assigned the scores. The prioritization criteria were taken from the CHNRI’s methodology (*answerability, effectiveness, deliverability, equity and potential impact on burden of mental disorders*) and were weighted by 43 people with diverse backgrounds [19].

In 2007, the Lancet Global Mental Health Group also reported the results of a prioritization exercise, using a similar methodology. The Lancet exercise was also conducted globally and focused on four groups of mental health conditions (i.e. common mental disorders, alcohol-use and other substance-abuse disorders, child and adolescent mental disorders, and psychotic disorders) [11]. Another prioritization exercise focusing specifically on mental health research priorities in 114 low- and middle income countries concluded that epidemiological studies of burden and risk factors, health systems research and social science research were of the highest priority [21].

In Europe, the ROAMER program [17] used a thorough methodology [22] to identify 151 research needs, and finally arrived at six overarching research priorities: prevention and promotion; causal mechanisms of mental health symptoms; development of international mental health research networks and databases; developing and implementing better interventions using new scientific advances; reducing stigma and empowering services users, and focusing on quality of care and sociocultural approaches in health systems [23].

The identified priorities differ somewhat from the Chilean ones, where eleven out of the top 13 priorities related to evaluation of the health care system and interventions. The cultural context as well as the health care system probably influence the perceived priorities. For example, there are fewer mental health resources in Chile, and their effective use in the public health care system is a priority. In a low-resource context, studying the causal mechanisms or investing in new technologies may not appear as immediately important objectives for the health care system. Also, the ROAMER methodology specifically asked respondents to assess priorities from an international, European point of view, not only their national needs.

Within Latin America, in 2010, the Brazilian Ministry of Health called for a mental health research prioritization study, using the CHNRI methodology. In the study, 28 experts from different fields identified 111 knowledge gaps. Similarly to the other studies, some members of the work group also prioritized 35 research questions [24]. Among the top ten priorities, four related to identification and treatment of mental disorders in primary care, and five to access, coverage and cost-effectiveness of different interventions used in public health care. There appears to be a similar focus on applied and health system oriented research among the Brazilian priorities as our results.

There has also been a specific effort to identify mental health research needs in humanitarian settings, and also in this exercise, half of the identified needs had to do with effectiveness and assessment of interventions [25].

In Chile, there has been one other experience prioritizing knowledge gaps, although it was not specific to mental health. The process included 34 individuals from various departments of the Ministry of Health and universities, who identified 11 major areas relevant to research. There was, however, no mention of the use of explicit prioritization criteria [26].

Regarding the quality of these studies, in low- and middle-income countries, research prioritization studies show high heterogeneity and few relevant actors—due in part to weak identification of stakeholders—as well as to lack of governmental leadership and no ongoing review of the prioritization process [14].

This situation also describes what has been observed in Latin America. Reveiz et al. explored a variety of sources, including official sites of governmental agencies, and identified 18 countries in the region with prioritization exercises. The authors indicate that there is little description of the context, or of the methodologies used. Furthermore, although 13 countries have documents that establish national health research priorities, only 6 provide specific research questions [15].

Our review of these experiences and other reports highlights at least three different methodological approaches to defining research priorities, where the previously mentioned CHNRI method [12] is one of the most widely used. Another approach corresponds to the Combined Approach Matrix (CAM), which, unlike CHNRI and our study, does not follow a standard prioritization process, but rather identifies knowledge gaps through a systematic and comprehensive process of gathering information, using a multi-dimensional matrix [27]. A third method, proposed by the Council on Health Research and Development (COHRED), includes a series of steps, departing from the evaluation of the initial situation and ending with the processes that assure the utilization of the outcomes by the policy makers. This approach allows for the use of the most appropriate methodological approach (including CHNRI and CAM), according to the needs, characteristics, and context of each prioritization exercise [28].

### Strengths and limitations

Comparing our study with the mentioned methodologies, and other existing reports, we should recognize some shortcomings. The first corresponds to the lack of a broader advisory committee to score each knowledge gap. This scoring process could be carried out independently by each member, with time set aside to discuss only the questions with varied scores.

Second, we use the same weight for the five prioritization criteria, while other studies have used participatory strategies to establish different weights for each criterion [19]. Nevertheless, further exploration of our data revealed that differential weighting of the criteria would not have substantially modified our results.

We did not include the feasibility of the research questions in our prioritization criteria, nor did we consider the ethical aspects of the questions, as has been done in other studies [12, 19], because those criteria did not emerge during the consultations with key informants or focus groups.

A third limitation corresponds to not carrying out a systematic review of the evidence to determine the level of knowledge, the magnitude of potential benefit, and the size of the population potentially affected, for each research question. We believe that this aspect should be included in future prioritizations, following international recommendations [13, 29].

In addition, it could be considered a limitation that the groups involved in the formulation of knowledge gaps (decision makers, users, caregivers, clinicians and researchers) did not work together at any time. Creating a horizontal discussion around the knowledge gaps was considered methodologically too complex. Recently, an interesting prioritization exercise grouped mental health professionals, users and carers, who developed somewhat different conclusions than those efforts led by health professionals only, emphasizing the need for stigma reduction and education [30].

Lastly, our study did not explicitly use a forward looking projection in the time frame to identify knowledge gaps, as recommended by some guidelines [13], although this could have been implicitly underlying in the conversations with participants during the process.

Among the study’s strengths, we note that this is the first in Chile to use a structured methodology to identify knowledge gaps, comparable to that used in international literature. We strived to use a methodology transparent in each stage and in the results, as well comprehensive and participatory [13, 23]. The process included consultations with key informants, in decision-making positions on mental health public policies, along with focus groups with service users and clinicians, and an open web consultation for academics. We also extracted contents from national relevant policy documents as well as from clinical practice guideline updating process. The prioritization criteria were all collected in a participatory manner, using the aforementioned interviews, to identify relevant local criteria [13, 23].

## Conclusions

This prioritization exercise identified and prioritized 155 knowledge gaps relevant to mental health policy decision-making through a systematic, transparent, and participatory process. As a result of this study, in 2014, the National Commission for Scientific and Technological Research (CONICYT, for its Spanish acronym) held its first Mental Health specific call for research proposals, based on the prioritized knowledge gaps (www.conicyt.cl/fonis/).

Prioritizing knowledge gaps should be an ongoing process [13]. The source that provided the greatest number of research questions was the online platform, which we could being used on a continuous basis, maintaining an open access for interested respondents. In addition to being used to disseminate the prioritized knowledge gaps—one of the principles of good practice for this type of process [13]—the online platform will enable knowledge exchange between investigators, who are currently working on priority issues. The other strategies of the exercise should be repeated regularly, to ensure the continued participation of service users and clinicians. Recently, calls have been made to involve the public and services users in the prioritization processes [30, 31].

We believe that this work represents an important advancement in the alignment of research with the health decision-making process in the country, and urge other sectors of health care as well as other countries of the region to replicate the process.

## Additional file

**Additional file 1.** Table S1 presents the number of people that participated in the data collection process, Table S2 Prioritization criteria and rankings, Table S3 Complete list of prioritized research questions.
