# Supplementary material for: Priority setting for mental health research in Chile
Source: Int J Ment Health Syst. 2017 Oct 2;11:61. doi: 10.1186/s13033-017-0168-9 (PMC5625763; doi:10.1186/s13033-017-0168-9)
Supplement: Supplementary file 1 — Additional file 1. Table S1 presents the number of people that participated in the data collection process, Table S2 Prioritization criteria and rankings, Table S3 Complete list of prioritized research questions. [file 13033_2017_168_MOESM1_ESM.doc]

**Supplementary material 1. Translation and adaptation of the Guides used in Semi-structured Interviews and Focus Groups.**

**Presentation**

We thank you in advance for your participation. As we mentioned in the invitation, we are interested in identifying the major gaps in mental health knowledge that are important to overcome to improve mental health for Chileans. According to this we created this exercise that should help us to achieve this purpose.

- pass the agreement for participating and being recorded.

**Awareness exercise**

Please, imagine that you are part of a scientific committee that was commanded by the Ministry of Health to select the 10 most important research questions whose answers should help to decide the investment on Mental Heath during the next 10 years

- table with questions is shown -

1. Which three of them do you consider are the most important questions that should be answered for orienting the investment on Mental Heath during the next 10 years?

- provide 5 – 10 minute to discuss the exercise.

1. Which three of them do you consider are less important questions that should be answered for orienting the investment on Mental Heath during the next 10 years?

- provide 5 – 10 minute to develop the exercise.

- in the case of focus group, each member should present their results.

**Identification of prioritization criteria**

1. Could you tell us which criteria did you use for selecting the more prioritized questions?

- provide 5 – 10 minute to discuss the question.

1. Why do you consider that these criteria are the most important?

- in the case of focus group, the question “d” is replaced by “From all of these criteria, which of them should be the most important, and why?”. It is an open question to the whole group.

**Proposal of significant knowledge gaps, related to mental health decision-making**

1. From your own experience, can you add five more knowledge gaps that you consider significant for the process of mental health decision-making?

- provide 10 – 15 minute to discuss the question.

- the moderator should try to clarify the components required for build the research question: “people“ ,“exposition”, “comparator” [if procedure], “outcome”.

- in the case of focus group, the answers are written on a board, allowing everybody to see the list.

**Close the meeting**

We thank you again for you unavailable help in this process. Remember that in in case of any question you can contact us….

**Link to the online questionnaire in Spanish:**

<https://docs.google.com/forms/d/1i9TZyQa3he0VTEbRiG5HnCwo5IGUnNn3vFktoc-7hxs/viewform?edit_requested=true>

**Supplementary Table 1. Number of people that participated in the data collection process.***

| **Source od data** | **Number of people** |
| --- | --- |
| Document analysis | 7 |
| Semi-structured interviews | 6 |
| Focus groups (3 groups) | 18 |
| On-line survey to academics | 23 |

* Additionally 63 people participated in the process of up-date of clinical practice guidelines, and also were indirectly involved in the process of knowledge gaps identification.

**Supplementary Table 2. Prioritization Criteria and Rankings**

| **Extent of Knowledge Gap** | |
| --- | --- |
| 1 | It is assumed that there is practically no information on the topic or related topics that would allow a sufficient approximation to the response of the research question in the local context. |
| 2 | It is assumed that there is partial information on the topic or related topics that would allow a sufficient approximation to the response of the research question in the local context. |
| 3 | It is assumed that there is abundant information on the topic or related topics that would allow a sufficient approximation to the response the research question in the local context. |
|  | |
| **Size of population directly benefiting from knowledge** | |
| 1 | The response to the question will directly benefit the entire population or almost the entire population |
| 2 | The response to the question will directly benefit a large part of the population, but not the entire population. |
| 3 | The response to the question will directly benefit a small portion of the population. |
|  | |
| **Vulnerability of population directly benefiting from knowledge** | |
| 1 | The response will directly benefit a clearly vulnerable section of the population. |
| 2 | The response will directly benefit a section of the population, with certain vulnerability. |
| 3 | The response will not directly benefit any vulnerable section of the population. |
|  | |
| **Magnitude of individual risk or benefit** | |
| 1 | The intervention or factor of the question represents a potentially great benefit or risk for the exposed individuals. |
| 2 | The intervention or factor of the question represents a potentially medium-sized benefit or risk for the exposed individuals. |
| 3 | The intervention or factor of the question represents a potentially small benefit or risk for the exposed individuals. |
|  | |
| **Urgency** | |
| 1 | The response to the question is urgently required by health authorities. |
| 2 | The response to the question is required with moderate urgency by health authorities. |
| 3 | The response to the question is not urgent for health authorities. |
|  | |
| **Applicability** | |
| 1 | The response to the question is directly applicable to decision-making related to public health policies. |
| 2 | The response to the question is partially applicable to decision-making related to public health policies, and possible requires translational research. |
| 3 | The response to the question is not directly applicable to decision-making related to public health policies, and requires more translational research. |

**Supplementary Table 3. Complete list of prioritized research questions**

| **Question** | **Source** | **Category** | **Score** |
| --- | --- | --- | --- |
| What are the determining factors of the variability in the comprehensive mental health care program, between primary care centers? | NHS | Evaluation of System and/or Health Program | 83.3 |
| What are the determining factors of the variability in the performance of primary health care human resources in the detection and management of mental disorders? | Key Informant | Evaluation of System and/or Health Program | 83.3 |
| What are the determining factors of the unchanging and unequal geographic distribution of specialized mental health human resources? | Users | Evaluation of System and/or Health Program | 75.0 |
| What is the prevalence of mental disorders in minority populations (ethnic groups, immigrants, homeless individuals)? | NHS | Natural History of Disease/Epidemiology/Risk Factors/Social Determinants | 70.0 |
| What is the prevalence of mental disorders and psychiatric comorbidity in the general population? | NHS | Natural History of Disease/Epidemiology/Risk Factors/Social Determinants | 70.0 |
| What is the effective coverage of interventions designed to manage mental disorders in affected individuals? | NHS | Evaluation of System and/or Health Program | 70.0 |
| What is the degree of integration (development) of the community model in mental health care facilities? | NCPPMD | Evaluation of System and/or Health Program | 70.0 |
| What is the variability of the performance of psychologists that work in the comprehensive mental health care program in primary care centers? | NHS | Evaluation of System and/or Health Program | 70.0 |
| What is the provision of mental human resources, according to care level and geographic location? | NHS | Evaluation of System and/or Health Program | 70.0 |
| What is the degree of adherence to the CPG recommendations for patients in the GES (universal, guaranteed treatment access) program for the management of schizophrenia, depression, and harmful alcohol and drug use? | NHS | Evaluation of System and/or Health Program | 70.0 |
| What are the transgenerational factors associated with intrafamilial child sexual abuse? | Directors of Clinical Services | Natural History of Disease/Epidemiology/Risk Factors/Social Determinants | 66.7 |
| What is the incremental cost-effectiveness ratio of evidence-based interventions to prevent adolescent suicide? | Online Consultation | Evaluation of Interventions | 66.7 |
| Is there bioequivalence between original and generic drugs used to manage depression? | Key Informant | Evaluation of Interventions | 66.7 |
| What factors determine the performance of mental health facilities in the different levels of care? | Key Informant | Evaluation of System and/or Health Program | 66.7 |
| What are the determining factors of compliance to the Clinical Practice Guidelines (CPG) of schizophrenia, depression, and harmful alcohol and drug use? | NHS | Evaluation of System and/or Health Program | 66.7 |
| What is the cost-effectiveness ratio of the psychosocial activities in municipal, educational, and work settings in the general population? | Key Informant | Evaluation of Interventions | 66.7 |
| What is the coverage of screening interventions and case management for suicide attempts in the general population? | Key Informant | Evaluation of System and/or Health Program | 66.7 |
| What factors influence the performance of the community model in different mental health care facilities? | NHS | Evaluation of System and/or Health Program | 66.7 |
| What is the effectiveness of the community model for mental health care, compared to the traditional care model in the general population? | NHS | Evaluation of System and/or Health Program | 66.7 |
| What are the financial, administrative, and regulatory opportunities involved in scaling up effective interventions to improve the mental health of individuals using the country’s various health facilities? | Key Informant | Evaluation of Intersectoral Public Policies | 60.0 |
| What mental health competencies must primary care health professionals have to ensure the effective functioning of the mental health care model? | Key Informant | Evaluation of System and/or Health Program | 60.0 |
| What is the variability in the performance of primary care health professionals in the detection and management of mental disorders? | Key Informant | Evaluation of System and/or Health Program | 60.0 |
| What is the validity and reliability of instruments used to evaluate the capacity to consent of individuals with a mental disorder? | NCPPMD | Evaluation of Interventions | 60.0 |
| What proportion of mental health care facilities comply with the mental health Clinical Practice Guidelines? | Key Informant | Evaluation of System and/or Health Program | 60.0 |
| What is the reliability of mental health information that is routinely collected by the DEIS [Department of Health Statistics and Information] (for example, data from the monthly statistical updates on hospital discharges)? | Key Informant | Evaluation of System and/or Health Program | 60.0 |
| What is the level of user satisfaction in the general population, with respect to mental health interventions and policies? | NCPPMD | Evaluation of System and/or Health Program | 60.0 |
| What is the additional cost for each unit increase in the effective coverage of mental health care? | Key Informant | Evaluation of System and/or Health Program | 60.0 |
| What is the additional cost for each unit increase in the mental health care coverage? | Key Informant | Evaluation of System and/or Health Program | 60.0 |
| What is the incidence of child sexual abuse? | Directors of Clinical Services | Natural History of Disease/Epidemiology/Risk Factors/Social Determinants | 60.0 |
| What is the burden and cost of caring for individuals with a mental disorder who should receive health care coverage through their job, in a separate network for workers, but are treated in the public system? | Directors of Clinical Services | Evaluation of System and/or Health Program | 60.0 |
| What factors determine the remission of major depressive episodes in patients treated in secondary level health services through the GES program, at 6 months, 1 year, and 2 years? | Online Consultation | Evaluation of System and/or Health Program | 58.3 |
| For women who are positively screened for postpartum depression with the EPDS during their postnatal care, what factors are associated with a decrease in symptomatology? | Online Consultation | Natural History of Disease/Epidemiology/Risk Factors/Social Determinants | 58.3 |
| What factors determine whether of not an interventions will decrease the disability of individuals with a mental disorder who are treated in mental health care facilities? | NCPPMD | Evaluation of System and/or Health Program | 58.3 |
| What are effective interventions that focus on psychosocial determinants in the promotion of child and adolescent mental health? | Directors of Clinical Services | Evaluation of Interventions | 58.3 |
| What are the most effective interventions to reduce disability in individuals with a mental disorder? | NCPPMD | Evaluation of Interventions | 58.3 |
| What is the validity and reliability of the Conners Test to detect ADHD in children and adolescents, when applied by parents and teachers in Chile? | Development of CPG | Evaluation of Interventions | 58.3 |
| What is the cost-benefit ratio of having general practitioners treat individuals with a mental disorder compared with treatment by a specialized mental health physician? | Department of Mental Health | Evaluation of Interventions | 58.3 |
| For adolescent law infractions, what is the fraction attributable to the health sector? | Online Consultation | Natural History of Disease/Epidemiology/Risk Factors/Social Determinants | 58.3 |
| What is the effectiveness of Open Social Networks (use of networks and thematic groups) in the presentation, promotion, and prevention of mental health problems in the general population? | Online Consultation | Evaluation of Intersectoral Public Policies | 58.3 |
| What is the effectiveness of intersectoral policies in the resolution of mental health problems and social inclusion, for children and adolescents? | Online Consultation | Evaluation of Intersectoral Public Policies | 58.3 |
| What is the effectiveness of interventions focused on reducing the stigmatization of individuals with a mental disorder by the general population? | Users | Evaluation of Intersectoral Public Policies | 58.3 |
| What is the effectiveness of interventions to prevent mental disorders in children and adolescents? | Online Consultation | Evaluation of Interventions | 58.3 |
| What is the validity and reliability of clinical screening instruments that are applied to individuals treated in the GES program for depression, schizophrenia, and harmful alcohol and drug use? | NHS | Evaluation of Interventions | 58.3 |
| What is the effectiveness and cost-effectiveness ratio of educational interventions, which aim to promote mental health in students? | NHS | Evaluation of Intersectoral Public Policies | 58.3 |
| What is the effectiveness and cost-effectiveness ratio of interventions in the workplace, which aim to promote mental health in the workforce? | NHS | Evaluation of Intersectoral Public Policies | 58.3 |
| What is the effectiveness and cost-effectiveness ratio of interventions in areas of public transportation, which aim to promote mental health in the general population? | NHS | Evaluation of Intersectoral Public Policies | 58.3 |
| What is the effectiveness of maternal communication with a history of child sexual abuse to her child in the prevention of transgenerational Child Sexual Abuse? | Directors of Clinical Services | Evaluation of Interventions | 58.3 |
| What is the coverage of interventions for family members of people who commit suicide? | Key Informant | Evaluation of System and/or Health Program | 58.3 |
| What are the mechanisms that affect the association between mental disorders and physical comorbidity in the general population (in terms of prevention)? | NHS | Natural History of Disease/Epidemiology/Risk Factors/Social Determinants | 50.0 |
| What factors present in the mental health network hinder the social inclusion of individuals with mental disabilities? | Online Consultation | Evaluation of Intersectoral Public Policies | 50.0 |
| What factors facilitate the adequate implementation of new mental health care programs in primary care center? | Key Informant | Evaluation of System and/or Health Program | 50.0 |
| What factors are associated with the successful incorporation of the mental health care program in primary care? | Department of Mental Health | Evaluation of System and/or Health Program | 50.0 |
| What is the validity and reliability of instruments used to measure the treatment evolution of adolescents between 10 and 14 years of age with depression? | Development of CPG | Evaluation of Interventions | 50.0 |
| What is the validity and reliability of instruments used to give a comprehensive evaluation of drug use in adolescents? | Development of CPG | Evaluation of Interventions | 50.0 |
| What is the validity and reliability of instruments used to screen for depression in adolescents between 10 and 14 years of age? | Development of CPG | Evaluation of Interventions | 50.0 |
| What is the cost-effectiveness ratio of the current treatment programs for harmful alcohol and drug use? | Online Consultation | Evaluation of System and/or Health Program | 50.0 |
| What is the cost-effectiveness ratio of interventions that aim to improve treatment adherence of individuals with a mental disorder? | Online Consultation | Evaluation of Interventions | 50.0 |
| What is the prevalence of mental disorders and their psychiatric comorbidity in children and adolescents? | NHS | Natural History of Disease/Epidemiology/Risk Factors/Social Determinants | 50.0 |
| What percentage of individuals with a mental disorder, that are treated in the public system, participate in a scheduled activity (work, schooling, clubs/groups, etc.)? | Key Informant | Evaluation of System and/or Health Program | 50.0 |
| What is the effectiveness of interventions that aim to prevent depression in the general population? | Online Consultation | Evaluation of Interventions | 50.0 |
| What is the effectiveness of the mental health network’s strategies to promote labor inclusion for individuals with a mental disability? | Online Consultation | Evaluation of Intersectoral Public Policies | 50.0 |
| What is the effectiveness of social and work inclusion strategies in mental health rehabilitation programs? | Online Consultation | Evaluation of Intersectoral Public Policies | 50.0 |
| What impact do intersectoral mental health policies have on the social reinsertion of individuals with mental disorders? | Department of Mental Health | Evaluation of Intersectoral Public Policies | 50.0 |
| What is the effectiveness of training primary health care professionals to detect and manage mental disorders? | Key Informant | Evaluation of System and/or Health Program | 50.0 |
| What is the effectiveness of interventions carried out in young populations to improve the mental health of those individuals as adults? | Key Informant | Evaluation of Interventions | 50.0 |
| What is the effectiveness of interventions for adolescents with risk behaviors, in terms of improving their short-term mental health? | Key Informant | Evaluation of Interventions | 50.0 |
| What is the countrywide effectiveness of pharmacological treatments for Attention Deficit Disorder in the population currently in treatment? | Online Consultation | Evaluation of Interventions | 50.0 |
| What is the difference in the performance of COSAM [community mental health centers] and other secondary level care facilities (CESAM), according to administrative unit (Health Service versus Municipality)? | Online Consultation | Evaluation of System and/or Health Program | 50.0 |
| What is the gap in human and physical health resource allowance in the community mental health facilities? | NCPPMD | Evaluation of System and/or Health Program | 50.0 |
| What is the knowledge gap in primary care health professionals about adequate management of mental disorders? | Key Informant | Evaluation of System and/or Health Program | 50.0 |
| To what degree is inter-partner violence detected in patients that consult general practitioners for general morbidity? | Online Consultation | Evaluation of System and/or Health Program | 50.0 |
| What is the degree of compliance to the standards of the human rights convention for the treatment of individuals with a mental health disability? | Online Consultation | Evaluation of System and/or Health Program | 50.0 |
| What is the most effective model of financial incentive (clinical improvement, population in treatment) in the delivery of mental health care in the various types of facilities? | Key Informant | Evaluation of System and/or Health Program | 50.0 |
| What is the impact of mental disorders and their psychiatric comorbidity in the general population (for example, economic)? | NHS | Natural History of Disease/Epidemiology/Risk Factors/Social Determinants | 50.0 |
| What is the impact of the different work styles used by mental health human resources? | Directors of Clinical Services | Evaluation of System and/or Health Program | 50.0 |
| What is the cost of different mental health service offerings? | Key Informant | Evaluation of System and/or Health Program | 50.0 |
| How does the deficit and high turnover of primary care physicians affect the effectiveness of the mental health care model? | Online Consultation | Evaluation of System and/or Health Program | 50.0 |
| What are the resources, competencies, and performance of mental health intervention teams, at the various levels of the network? | Directors of Clinical Services | Evaluation of System and/or Health Program | 50.0 |
| What factors are associated with the involuntary hospitalizations of individuals with a mental disorder? | Directors of Clinical Services | Evaluation of System and/or Health Program | 50.0 |
| What are the gaps in child-adolescent mental health promotion activities, focused on psychosocial determinants? | Directors of Clinical Services | Evaluation of Intersectoral Public Policies | 50.0 |
| Is the enrollment of individuals into GES programs for a mental health pathology in line with the established criteria? | Directors of Clinical Services | Evaluation of System and/or Health Program | 50.0 |
| What is the magnitude and type of disability of individuals in treatment for schizophrenia in the health care network? | Directors of Clinical Services | Evaluation of System and/or Health Program | 50.0 |
| What infrastructure (capacity/beds in sheltered homes, day hospitals, short-stay hospitals) is available for mental health treatment? | NHS | Evaluation of System and/or Health Program | 50.0 |
| What is the adherence of individuals with mental disorders to the various types of therapeutic interventions? | NHS | Evaluation of Interventions | 50.0 |
| For individuals with first-episode schizophrenia, what is the effectiveness of early interventions and secondary prevention? | Online Consultation | Evaluation of System and/or Health Program | 41.7 |
| What variables determine the effectiveness of rehabilitation programs for individuals with severe mental disorders? | Online Consultation | Evaluation of System and/or Health Program | 41.7 |
| Are there better payment mechanisms than the DRG (diagnostic-related group) system or the PAD (payment related to a diagnostic) method of the AUGE program for mental illnesses, according to the international literature and experiences? | Online Consultation | Evaluation of System and/or Health Program | 41.7 |
| What are the predictors of work-related mental health problems (in the workforce)? | Key Informant | Natural History of Disease/Epidemiology/Risk Factors/Social Determinants | 41.7 |
| What are the determinants of violent behavior in individuals with a mental disorder, attributable or not to their condition? | Directors of Clinical Services | Natural History of Disease/Epidemiology/Risk Factors/Social Determinants | 41.7 |
| What are the (modifiable) risk factors of suicidality in adolescents 15 to 19 years of age? | Online Consultation | Natural History of Disease/Epidemiology/Risk Factors/Social Determinants | 41.7 |
| What are the mental health human resource competencies related to the respectful treatment of individuals with mental disorders and their families? | Users | Evaluation of System and/or Health Program | 41.7 |
| What is the validity, reliability, and applicability of instruments that evaluate disability associated with a mental disorder in population surveys? | NHS | Evaluation of Interventions | 41.7 |
| What is the validity and reliability of instruments that detect bipolar disorder in the individuals attending health services? | Development of CPG | Evaluation of Interventions | 41.7 |
| What is the cost-effectiveness ratio of interventions (??) designed to prevent neurocognitive deficits of patients with bipolar disorder? | Development of CPG | Evaluation of Interventions | 41.7 |
| What is the effectiveness and cost-effectiveness ratio of sectoral mental health policies focused on the social reinsertion of individuals with a mental disorder, in terms of relapses and recurrences? | Department of Mental Health | Evaluation of Interventions | 41.7 |
| What is the effectiveness of standard rehabilitation programs versus community-based psychosocial rehabilitation programs for individuals with a mental illness-related disability? | Online Consultation | Evaluation of Interventions | 41.7 |
| What is the effectiveness of population-level populations aimed at socially integrating individuals with a severe mental disorder? | Users | Evaluation of Interventions | 41.7 |
| What is the effectiveness of ECT as a treatment for individuals with a bipolar disorder? | Development of CPG | Evaluation of Interventions | 41.7 |
| What is the effectiveness of interventions that aim to facilitate the use of public transportation by individuals with a severe mental illness, in terms of their overall functioning? | Users | Evaluation of Intersectoral Public Policies | 41.7 |
| What is the variability of treatment offered by different mental health professionals for individuals with a mental disorder, and what are the health consequences of this? | Online Consultation | Evaluation of System and/or Health Program | 41.7 |
| What is the effectiveness and cost-effectiveness ratio of housing and urban planning interventions designed to promote mental health in the general population? | NHS | Evaluation of Intersectoral Public Policies | 41.7 |
| What competencies do mental health professionals need to have in order to improve detection of bipolar disorder? | Development of CPG | Evaluation of Interventions | 40.0 |
| What rehabilitations services are offered for individuals with a mental disorder in the public and private health networks? | Key Informant | Evaluation of System and/or Health Program | 40.0 |
| What is the level of disability associated with specific mental disorders in the general population? | NHS | Natural History of Disease/Epidemiology/Risk Factors/Social Determinants | 40.0 |
| What is the incidence of violent acts toward third parties in the general population? | Key Informant | Natural History of Disease/Epidemiology/Risk Factors/Social Determinants | 40.0 |
| What is the frequency of different work styles among mental health teams? | Directors of Clinical Services | Evaluation of System and/or Health Program | 40.0 |
| What is the burden attributable to mental disorders among individuals with a chronic, non-communicable disease? | NHS | Natural History of Disease/Epidemiology/Risk Factors/Social Determinants | 40.0 |
| What is the level of knowledge and competencies of psychiatrists who complete residencies financed by the public health system, in terms of the programs and practices recommended by the National Mental Health Plan, the Explicit Health Guarantees Program (GES), the Law of Rights and Responsibilities of Patients, the International Conference on the Rights of People with Disability, the Hospital Admission Regulation, the Containment Standards, and other similarly important instruments? | Online Consultation | Evaluation of System and/or Health Program | 40.0 |
| How much does the Model of Comprehensive Care, with a family and community focus, complement the Model of Mental Health in primary health care service delivery? | Online Consultation | Evaluation of System and/or Health Program | 40.0 |
| What are the financial management models of the various mental health facilities? | Key Informant | Evaluation of System and/or Health Program | 40.0 |
| What is the degree of inclusion of individuals with a severe mental disorder in the various primary care programs (not only the mental health programs)? | Online Consultation | Evaluation of System and/or Health Program | 40.0 |
| How do different social groups, that are important to the country’s development (businessmen, workers, politicians, etc.), value child-adolescent mental health, and how is this interest converted into concrete actions? | Online Consultation | Evaluation of System and/or Health Program | 40.0 |
| What is the prevalence of different types of mental illness in the population with a neurological disorder from childhood? | Directors of Clinical Services | Natural History of Disease/Epidemiology/Risk Factors/Social Determinants | 40.0 |
| What is the incidence of suicide attempts in the general population of the country? | Key Informant | Natural History of Disease/Epidemiology/Risk Factors/Social Determinants | 40.0 |
| What is the health burden associated with psychosocial determinants of child-adolescent mental health? | Directors of Clinical Services | Natural History of Disease/Epidemiology/Risk Factors/Social Determinants | 40.0 |
| What are the psychosocial, socio-demographic, and familial factors that most determine the incidence of mental disorders in the general population? | Department of Mental Health | Natural History of Disease/Epidemiology/Risk Factors/Social Determinants | 33.3 |
| What work conditions are related to the respectful treatment of individuals with mental disorders and their families, by mental health professionals? | Users | Evaluation of System and/or Health Program | 33.3 |
| What is the cost-effectiveness ratio of interventions that aim to improve the quality of life of patients with an organic disease (e.g. mental retardation, developmental disorder with behavioral alterations)? | Online Consultation | Evaluation of Interventions | 33.3 |
| What is the effectiveness of the community mental health care model in the rehabilitation of individuals with dual diagnosis? | Online Consultation | Evaluation of System and/or Health Program | 33.3 |
| What is the effectiveness of complementary interventions (e.g. mediation) in the prevention of mental health problems in the general population? | Key Informant | Evaluation of Interventions | 33.3 |
| What is the impact of each additional appointment with a mental health specialist on the overall functioning of individuals with a severe mental disorder? | Users | Evaluation of Interventions | 33.3 |
| What factors are associated with the rehospitalization of individuals discharged from short-stay psychiatric hospitalizations? | Online Consultation | Evaluation of System and/or Health Program | 33.3 |
| What are the psychosocial determinants of child-adolescent mental health? | Directors of Clinical Services | Natural History of Disease/Epidemiology/Risk Factors/Social Determinants | 33.3 |
| What is the cost-effectiveness ratio of the application of traditional psychopathology criteria versus the criteria of the DSM-V/ICD-10 in the diagnosis and management of mental disorders? | Directors of Clinical Services | Evaluation of Interventions | 33.3 |
| What is the prevalence of mental disorders among mental health human resources, and what are the determinants? | Directors of Clinical Services | Natural History of Disease/Epidemiology/Risk Factors/Social Determinants | 33.3 |
| What is the impact of interventions that mental health professionals teach family members of individuals with mental disorders, on the patients’ overall functioning? | Users | Evaluation of Interventions | 33.3 |
| What impact do the mental health and personal issues of mental health center teams have on the outcomes of service users? | Directors of Clinical Services | Evaluation of System and/or Health Program | 33.3 |
| What percentage of the mental health investment budget is consistent with the current mental health policy? | Online Consultation | Evaluation of System and/or Health Program | 30.0 |
| What are the most frequent comorbidities affecting patients enrolled in the GES program for depression in primary care? | Online Consultation | Natural History of Disease/Epidemiology/Risk Factors/Social Determinants | 30.0 |
| What are the immediate causes of the functional dependence and cognitive deterioration of older adults? | Online Consultation | Natural History of Disease/Epidemiology/Risk Factors/Social Determinants | 30.0 |
| What should be the content of laws specifically related to mental health, throughout the world? | Key Informant | Evaluation of Intersectoral Public Policies | 30.0 |
| What is the prevalence of burnout among health human resources? | Key Informant | Natural History of Disease/Epidemiology/Risk Factors/Social Determinants | 30.0 |
| What is the availability of jobs for individuals with a psychic disability? | Key Informant | Evaluation of Intersectoral Public Policies | 30.0 |
| What is the social acceptance of, and degree of community participation in, psychosocial rehabilitation programs for individuals with a mental disorder? | Online Consultation | Evaluation of System and/or Health Program | 30.0 |
| What is the satisfaction level of direct and indirect mental health service users, with respect to child-adolescent mental health treatment? (note: “indirect” users are family members, for example) | Online Consultation | Evaluation of System and/or Health Program | 30.0 |
| What is the performance of psychosocial rehabilitation units in the public system mental health network? | Online Consultation | Evaluation of System and/or Health Program | 30.0 |
| What is the validity of the DSM-V/ICD-10 criteria with respect to traditional psychopathology criteria? | Directors of Clinical Services | Evaluation of Interventions | 30.0 |
| For individuals treated in outpatient psychiatric services, what factors are associated with a favorable evolution (early discharge, post-discharge evolution, readmission | Online Consultation | Evaluation of System and/or Health Program | 25.0 |
| What factors determine the adherence of individuals with a mental disorder to psychiatric treatment plans? | Online Consultation | Evaluation of System and/or Health Program | 25.0 |
| What are the long-term, adverse effects of psychotropic medication use? | Key Informant | Natural History of Disease/Epidemiology/Risk Factors/Social Determinants | 25.0 |
| What is the best way to finance community-based activities in mental health programs? | Online Consultation | Evaluation of System and/or Health Program | 25.0 |
| What is the most cost-effective method to manage the effects of trauma in patients seeking treatment for depression? | Online Consultation | Evaluation of Interventions | 25.0 |
| What is the quality of life of individuals with schizophrenia who receive treatment in the comprehensive mental health program (psychotropic medications, psychosocial interventions, etc.) versus that of individuals who receive usual care? | Online Consultation | Evaluation of Interventions | 25.0 |
| What impact does the GES depression program have on the incidence and duration of sick leave in the working population? | Department of Mental Health | Evaluation of Interventions | 25.0 |
| What is the effectiveness of psychosocial interventions in individuals treated for schizophrenia? | Directors of Clinical Services | Evaluation of Interventions | 25.0 |
| What is the impact of treatment on the health-related quality of life (HRQoL) and subjective wellbeing of individuals with a mental disorder? | Directors of Clinical Services | Evaluation of Interventions | 25.0 |
| What elements of mental functioning predict the occurrence of violence between individuals, at the population level? | Key Informant | Natural History of Disease/Epidemiology/Risk Factors/Social Determinants | 20.0 |
| What sources of intersectoral information could be used to inform clinical decision-making? | Key Informant | Evaluation of Intersectoral Public Policies | 20.0 |
| What conception of human rights do professionals carrying out mental health interventions have, and how is this expressed in care? | Online Consultation | Evaluation of System and/or Health Program | 20.0 |
| What is the frequency of inappropriate use of benzodiazepines and anti-depressants in the general population? | Key Informant | Natural History of Disease/Epidemiology/Risk Factors/Social Determinants | 20.0 |
| What is the comorbidity of individuals with severe mental disorders? | Online Consultation | Natural History of Disease/Epidemiology/Risk Factors/Social Determinants | 20.0 |
| What is the profile of the population treated in outpatient psychiatric services (polyclinics of psychiatric and general hospitals, diagnostic and treatment centers [CDT], rural health centers [CRS], COSAM, and CESAM)? | Online Consultation | Evaluation of System and/or Health Program | 20.0 |
| What is the cost-effectiveness ratio of GES program interventions for depression? | Online Consultation | Evaluation of Interventions | 16.7 |
| How large is the effect that stigma has on treatment adherence, among individuals with severe mental disorders? | Users | Evaluation of Interventions | 16.7 |
| What is the prevalence of pervasive developmental disorders in the adult population? | Directors of Clinical Services | Natural History of Disease/Epidemiology/Risk Factors/Social Determinants | 10.0 |
